# Ultra-low contrast agent dosage in photon-counting CT angiography of the thoracoabdominal aorta

**DOI:** 10.1016/j.ejro.2026.100777

**Published:** 2026-06-22

**Authors:** Jan-Lucca Hennes, Kristina Krompaß, Henner Huflage, Pauline Pannenbecker, Ramin Saam Dazeh, Jan-Peter Grunz, Viktor Hartung, Thorsten Alexander Bley, Philipp Gruschwitz

**Affiliations:** Department of Diagnostic and Interventional Radiology, University Hospital Würzburg, Würzburg, Germany

**Keywords:** Photon-counting CT angiography, thoracoabdominal aorta, contrast agent dose reduction, virtual monoenergetic imaging, renal impaired patients

## Abstract

**Purpose:**

To assess the viability of an ultra-low contrast agent protocol (ULC) for aortic photon-counting CT angiography (PCD-CTA).

**Materials and methods:**

For this retrospective, single-center study, 31 consecutive patients receiving PCD-CTA with 48 ml Imeron® 150mgI/ml (≙ 7.2 g iodine) were assessed. Virtual monoenergetic images with 45/55 keV were compared to a control sample scanned with radiation-dose-equivalent 55 keV protocols (120kVp; image-quality-level 42) employing standard (SC; 60 ml Imeron® 350mgI/ml ≙ 21 g iodine) and low contrast agent dose (LC; 40 ml Imeron® 350 mg/ml ≙ 14 g iodine). Luminal attenuation and image noise were measured, while contrast-to-noise ratios were calculated. Five radiologists rated overall image quality, luminal attenuation, and diagnostic confidence using a 5-point rating scale.

**Results:**

Radiation dose and body-mass-index did not differ significantly among groups (*p* > .999, each). ULC scans displayed significantly lower CNR (55 keV: 12.2 ± 2.7; 45 keV: 20.0 ± 4.6) than SC (35.3 ± 7.5) and LC studies (27.4 ± 10.7; each *p* ≤ .001) due to reduced luminal attenuation (ULC-55keV: 211.9 ± 27.3HU; ULC-45keV: 302.9 ± 42.8HU; SC 504.3 ± 70.9HU; LC: 380.8 ± 98.3HU; *p* < .001). Overall image quality of protocols was rated similar (each 4 [4−4]; *p* ≥ .395), while luminal attenuation of ULC-45keV (4 [4−4]) was rated inferior to SC (5 [5−5]; *p* < 0.001) but not to LC (4 [4−5]; *p* = 0.25). Diagnostic confidence was good for ULC (45 keV: 4 [4−4]); 55 keV: 3 [3−4]) and excellent for SC (5 [5−5]) and LC (5 [4−5]).

**Conclusion:**

Despite inferior contrast, aortic PCD-CTA with ultra-low contrast agent dosage at 45 keV allows for diagnostic image quality. The proposed scan protocol may hold potential for kidney protection in vulnerable patient groups.

## 1 Introduction

Despite the ongoing improvements in patient safety with regard to contrast-enhanced CT examinations, the risk of inducing nephropathy due to the application of iodinated contrast agent still poses a significant drawback in its use in clinicalpractice. [1]. The likelihood of developing contrast agent-induced nephropathy is elevated in patients with compromised kidney function and diabetes mellitus, and it is further augmented by the administration of hyperosmolar contrast medium [2]. Patients afflicted with aortic diseases, such as aneurysms or dissections, are particularly vulnerable for two reasons: Patients are repeatedly exposed to iodinated contrast agents because they require CT angiographies (CTA) for initial diagnosis, perioperative imaging, and follow-up [3]. Concurrently, renal insufficiency frequently occurs as a comorbidity of aortic diseases [4], [5], which has been demonstrated to elevate the risk of developing contrast agent-induced nephropathy [1].

In recent years, technological advances in the form of the photon-counting detector CT (PCD-CT) have been shown to facilitate a relevant reduction in required contrast agent dose in several vascular applications, such as CTA of the coronary or abdominal arteries [6], [7]. This detector technology converts the incoming X-ray photons directly into electrical signals, thereby enabling the measurement of their individual energy. The data obtained by this method contains spectral information that can be used for various purposes. First, it enables the elimination of electronic background noise and, consequently, the reduction of radiation dose [8]. Secondly, PCD-CTA can be utilized to enhance the contrast-to-noise ratio [9]. This, in turn, can lead to a reduction in the required contrast agent due to the higher weighing of low-energy photons while maintaining diagnostic image quality. According to extent evidence in literature, further reduction in the amount of contrast agent appears to be feasible by fully exploiting the new technology [10], [11], [12]. This approach may contribute to the preservation of renal function, particularly in patients with aortic diseases or pre-existing renal dysfunction.

The objective of this study was to assess the viability of an ultra-low contrast agent protocol (ULC) for PCD-CTA in comparison to the image quality of CT angiographies with reduced and standard contrast agent doses.

## 2 Materials and methods

### 2.1 Study design and patient sample

The local ethics committee approved this retrospective single-center study, which adheres to the principles of the Declaration of Helsinki in its most recent form. The requirement for additional written informed consent was waived (protocol number: 2025–125-dvhd). Consecutive patients receiving clinically indicated CTA of the thoracoabdominal aorta with a first-generation PCD-CT (Naeotom Alpha, Siemens Healthineers, Forchheim, Germany) between August and October 2024 were included in the study. The exclusion of patients from the study was based solely on deviations from the established protocols for CT scans or contrast agent administration, with a threshold of > 2 ml contrast agent volume.

### 2.2 Contrast agent protocols

PCD-CTA were performed using an ULC contrast agent protocol, as the institutional standard during the study period. The examinations were conducted with a volume of 48 ml of low-concentrated iodine-containing contrast agent (Imeron® 150 mg iodine/ml; Bracco, Milan, Italy; corresponds to 7.2 g iodine) administered with a flow rate of 2.5 ml/s via a 20-gauge peripheral venous catheter, resulting in a iodine delivery rate (IDR) of 0.375 gI/s. Administration included a pre- and post-bolus chaser of 20 ml saline solution using an automatic injector (CT motion, Ulrich GmbH, Ulm, Germany). For bolus tracking, a region of interest (ROI) was positioned within the descending thoracic aorta with a fixed trigger threshold (an increase of 80 HU), and a post-trigger delay of 7 s, in order to ensure optimal arterial contrast. The ULC protocol was compared with two previously clinical established protocols: a) a standard contrast agent protocol (SC) using 60 ml of conventional contrast agent (Imeron® 350 mg iodine/ ml; corresponding to 21.0 g of iodine) and b) a low contrast agent dose protocol (LC) using 40 ml of the identical contrast agent (corresponding to 14.0 g of iodine), both with a flow rate of 2.5 ml/s as well, corresponding to IDRs of 0.875 gI/s. Contrast agent protocols are summarized in Table 1.

**Table 1:** Contrast agent protocols.

	SC	LC	ULC
Iodine concentration (mgI/ml)	350	350	150
Contrast agent volume (ml)	60	40	48
Flow rate (ml/s)	4.0	4.0	2.5
Total iodine application (g)	21.0	14.0	7.2
Iodine delivery rate (gI/s)	1.4	1.4	0.375

SC, standard contrast agent dose protocol; LC, low contrast agent dose protocol; ULC, ultra-low contrast agent dose protocol.

### 2.3 Image acquisition and reconstruction

PCD-CTA procedures were executed as ECG-triggered high-pitch dual-source scans in a cranio-caudal direction with a pitch factor of 3.2 and a rotation time of 0.25 s. For data acquisition, the multi-energy mode (Quantum plus, Siemens) with a fixed tube voltage of 120 kVp and an image quality level of 42 in standard acquisition mode with 2 × 2 pixel binning and a collimation of 144 × 0.4 mm was utilized. Raw data was reformatted using a soft vessel-optimized reconstruction kernel (Bv36), with a slice thickness of 3 mm and an increment of 3 mm as virtual monoenergetic images (VMI) at 55 keV for ULC, LC, and SC. Furthermore, ULC scans were reconstructed as 45 keV VMI. The field of view was pre-set at 368 × 368 mm and only adapted if necessary, depending on the subject’s abdominal girth. The pixel matrix underwent automatic adaptation by the scanner, expanding from 512^2^ to 768^2^ pixels depending on the selected FOV.

### 2.4 Quantitative image comparison

Attenuation within the arterial lumen (HU_artery_) was measured by placing ROIs at five distinct levels: the ascending aorta at the level of the pulmonary trunk, the aortic arch, the descending thoracic aorta at the level of the right atrium, the descending abdominal aorta at the level of the celiac trunk, and the left or right common iliac artery, depending on luminal diameter. ROIs were placed on the respective slices in muscle and fat tissue, whereby the standard deviation of the Hounsfield units (SD_fat_) was determined to be representative of image noise. Furthermore, on each slice where intraluminal attenuation was measured, a region of interest (ROI) was placed in both muscle and adipose tissue to measure attenuation in muscle and the standard deviation of attenuation in adipose tissue, the latter serving as a measure of image noise. The values obtained were then utilized to calculate the mean values for intraluminal attenuation (HU_artery_), attenuation in muscle (HU_muscle_), and image noise (SD_fat_). These mean values were then employed for further analysis. Representative sample images with designated ROIs can be found in Figure S1 of the Supplementary Materials. Table S1 also provides a detailed list of the measurements. Contrast-to-noise ratios (CNR) were calculated using the following formula:

$$\;\mathrm{CNR}=\;\frac{\left(\mathrm{HUartery}-\mathrm{HUmuscle}\right)}{\mathrm{SDfat}}.$$

### 2.5 Qualitative image comparison

The assessment of image quality of PCD-CTA was conducted independently by three radiology residents with two to three years of experience in cardiovascular imaging, and two board-certified radiologists with seven and nine years of experience in cardiovascular imaging. The observers were blinded to the contrast agent protocols using pre-set hangings in the picture archiving and communication system. The subjective assessment encompassed overall image quality, lumen attenuation, and diagnostic confidence using a 5-point rating scale (1 = poor; 5 = excellent) with each category being evaluated individually.

### 2.6 Statistical analysis

The statistical analysis was conducted using specialized software (numiqo e.U., Graz, Austria). The normality of the metric data was confirmed through the implementation of Kolmogorov-Smirnov tests. For continuous variables with a normal distribution, the mean value and standard deviation are reported. In instances where the variable is non-continuous, the median value and interquartile range are reported. The objective image quality parameters were evaluated using a parametric one-way analysis of variance without repeated measures. For subjective image quality parameters, the ratings of the five observers were aggregated and a non-parametric Kruskal-Wallis test was performed. The statistical significance of the findings is established at p < 0.05. Kendall's W coefficient was employed to evaluate interrater reliability. Coefficients less than 0.5 were deemed to signify inadequate agreement. Those ranging from 0.5 to 0.8 were classified as good agreement, and those exceeding 0.8 were designated as excellent agreement [13].

## 3 Results

### 3.1 Patient characteristics

**Fig. 1:**
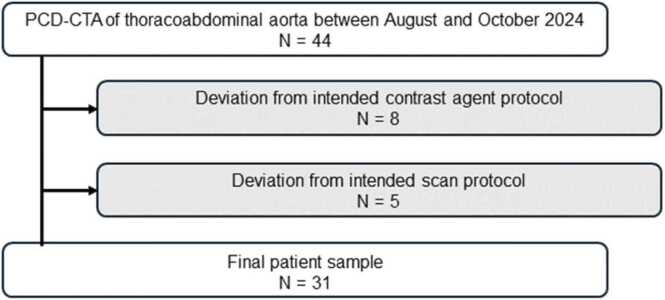
Flowchart visualizing patient exclusion criteria and the final study sample.**,***PCD-CTA, photon-counting detector computed tomography angiography; N = number of patients**.***

A total of 44 CTA examinations employing the ULC contrast agent protocol were conducted during the study period. Thirteen patients were excluded from the study due to deviations from the contrast agent protocol (n = 8) and CT scan protocol (n = 5). The final study sample comprised 31 patients (11 female). A flow chart illustrating inclusions and exclusions is given in Fig. 1. Patients who received the ULC protocol were compared to an equal number of body-mass-index matched patients examined using LC or SC protocol. An analysis of the mean age of the patients revealed no significant differences among the distinct subgroups (ULC: 67 ± 11 years; SC: 66 ± 8 years and LC: 61 ± 14 years; p ≥ 0.230). Similarly, no statistically significant difference was observed in the body-mass-index (ULC: 26.0 ± 4.8 kg/m^2^; LC 26.2 ± 4.3 kg/m^2^; SC: 27.4 ± 4.6 kg/m^2^; p ≥ 0.810). The use of constant image quality levels across all protocols yielded no statistically significant variation in the administered radiation doses (ULC: 2.83 ± 0.8 mGy; LC: 2.83 ± 0.8; SC: 3.12 ± 0.9 mGy; p ≥ 0.740). Table 2 presents a comprehensive overview of the patient characteristics.

**Table 2:** Patient characteristics.

	SC	LC	ULC
Age (years)	65.6 ± 8.2^a^	61.4 ± 14.3^a^	66.9 ± 10.6^a^
Weight (kg)	81.5 ± 14.0^a^	77.8 ± 15.3^a^	75.9 ± 12.5^a^
Height (m)	1.7 ± 0.1^a^	1.7 ± 0.1^a^	1.7 ± 0.1^a^
BMI (kg/m^2^)	27.4 ± 4.8^a^	26.2 ± 4.3^a^	26.0 ± 4.8^a^
CTDI_Vol_ (mGy)	3.1 ± 0.9^a^	2.8 ± 0.8^a^	2.8 ± 0.8^a^
SSDE (mGy)	3.6 ± 0.7^a^	3.4 ± 0.6^a^	3.4 ± 0.7^a^
DLP	212.4 ± 52,1^a^	200.9 ± 53.8^a^	193.5 ± 47.16^a^

Values are given as mean ± standard deviation. No significant differences were observed between the three contrast agent protocols for any of the parameters reported.

SC, standard contrast agent dose protocol; LC, low contrast agent dose protocol; ULC, ultra-low contrast agent dose protocol.

BMI, body mass index; CTDI_Vol_, volume CT dose index; SSDE, size specific dose estimate; DLP, dose length product.

a No significant differences between the groups (*p≥*0.05)

### 3.2 Quantitative image comparison

**Fig. 2:**
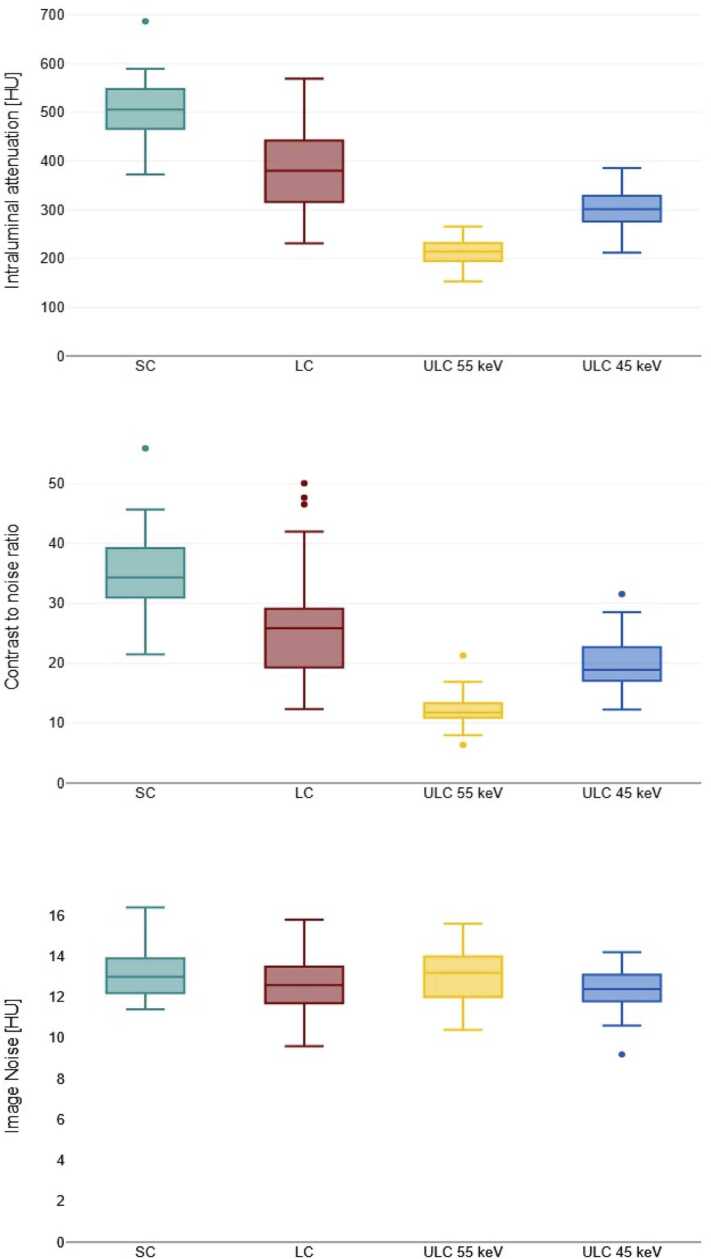
Boxplot diagrams summarizing the intraluminal attenuation, image noise and contrast-to-noise ratios when employing the different protocols.**,***SC, standard contrast agent dose protocol; LC, low contrast agent dose protocol; ULC, ultra-low contrast agent dose protocol; HU, Hounsfield units*.

Intraluminal attenuation was lowest in ULC 55 keV (211.9 ± 27.3 HU) and highest in SC (504.3 ± 72.6 HU; p < 0.001), followed by LC (380.8 ± 100.3 HU; p < 0.001). The 45 keV VMI of ULC (302.9 ± 42.8 HU) also exhibited lower attenuation compared to LC and SC (p < 0.001, respectively). However, ULC 45 keV yielded higher values than ULC 55 keV (p < 0.001). Image noise showed no substantial disparities among ULC 45 and 55 keV, LC, and SC (ULC 45 keV: 12.3 ± 1.2 HU; ULC 55 keV: 13.1 ± 1.3 HU; LC: 12.5 ± 1.6; SC: 13.1 ± 1.2 HU; each p ≥ 0.080). The lowest CNR value was calculated for ULC 55 keV (12.2 ± 2.7), while LC (27.35 ± 10.7; p < 0.001) and SC (35.3 ± 7.5; p < 0.001) provided markedly higher CNR values. The CNR of ULC 45 keV (20.0 ± 4.6) ranked between ULC 55 keV and LC (p < 0.010, respectively). The objective image quality parameters are summarized in Table 3. Luminal attenuation, image noise, and CNR are represented graphically as box plots in Fig. 2.

**Table 3:** Objective image quality.

	SC	LC	ULC 55 keV	ULC 45 keV
Luminal attenuation	504.3 ± 70.9^3^^,^^4^^,^^5^	380.8 ± 98.3^2^^,^^4^^,^^5^	211.9 ± 27.3^2^^,^^3^^,^^5^	302.9 ± 42.8^2^^,^^3^^,^^4^
Attenuation muscle	48.9 ± 4.7^5^	51.4 ± 5.7^5^	53.5 ± 5.8^5^	59.8 ± 13.3^2^^,^^3^^,^^4^
Image noise	13.1 ± 1.2^1^	12.5 ± 1.6^1^	13.1 ± 1.3^1^	12.3 ± 1.2^1^
Contrast-to-noise ratio	35.3 ± 7.5^3^^,^^4^^,^^5^	27.4 ± 10.7^2^^,^^4^^,^^5^	12.2 ± 2.7^2^^,^^3^^,^^5^	20.0 ± 4.6^2^^,^^3^^,^^4^

Except for contrast-to-noise ratios, values are given as mean ± standard deviation of Hounsfield units.

SC, standard contrast agent dose protocol; LC, low contrast agent dose protocol; ULC, ultra-low contrast agent dose protocol.

1 No significant differences between the groups (*p≥*0.05)

2 Significantly different compared to SC (*p* < 0.05)

3 Significantly different compared to LC (*p* < 0.05)

4 Significantly different compared to ULC 55 keV (*p* < 0.05)

5 Significantly different compared to ULC 45 keV (*p* < 0.05)

### 3.3 Qualitative image comparison

**Fig. 3:**
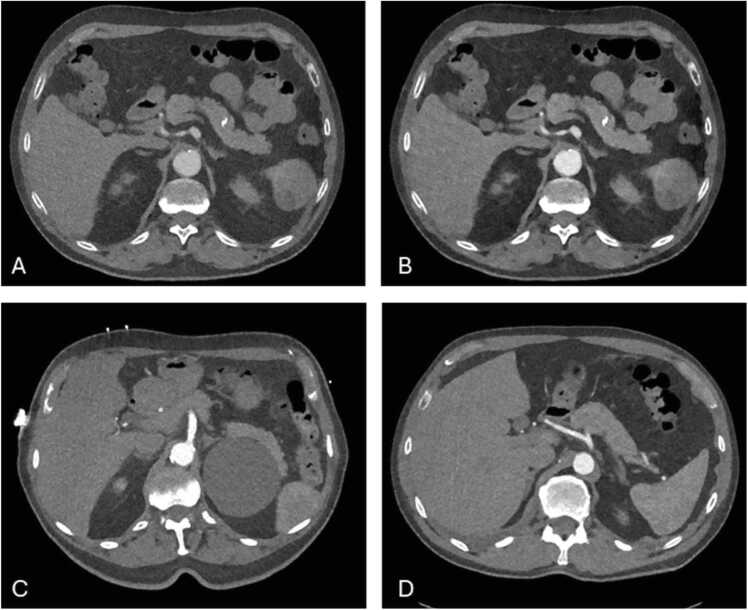
Photon-counting detector CT angiography in different patients with comparable body mass index. Images were generated with 55 keV ultra-low contrast agent dose (**A**), 45 keV ultra-low contrast protocol (**B**), standard contrast agent dose (**C**), and low contrast agent dose (**D**). Window settings: width 700 HU; center 100 HU.

**Fig. 4:**
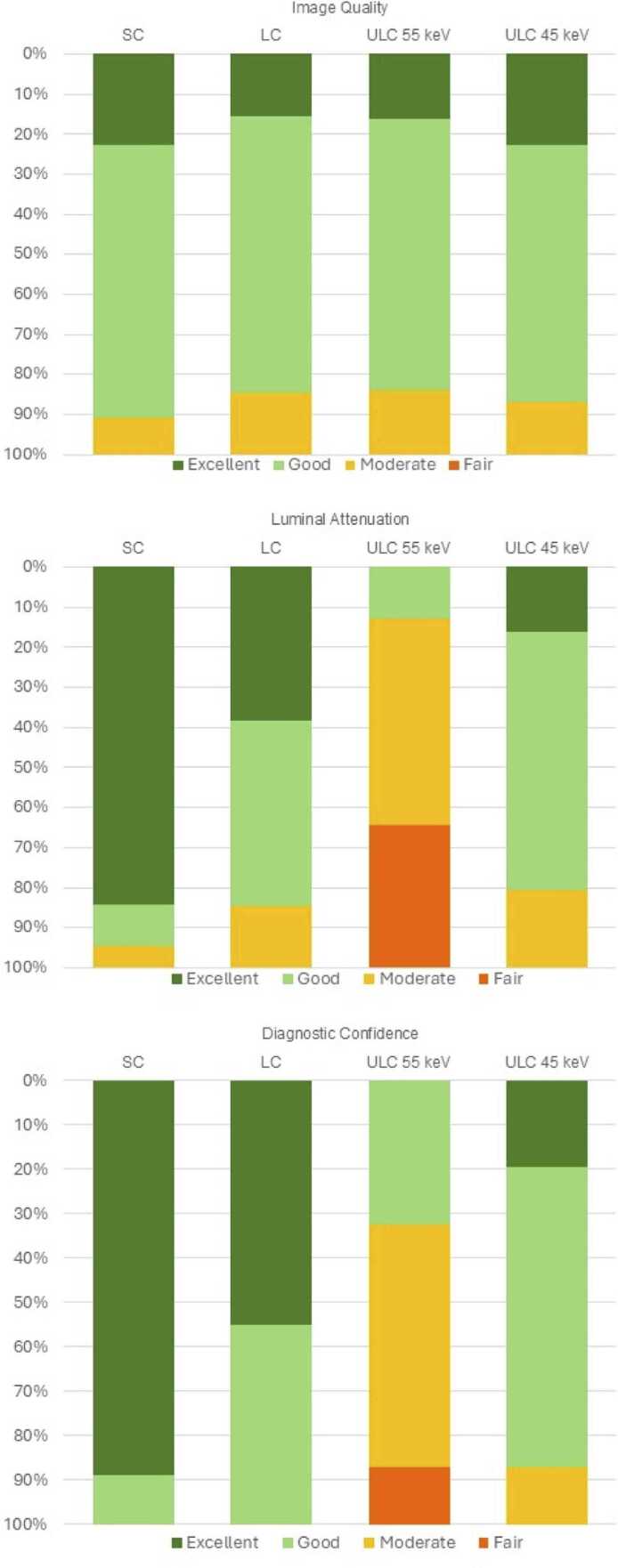
Subjective image quality scores for standard (SC), low (LC), and ultra-low (ULC) contrast agent protocols, the latter at 45 keV and 55 keV. Stacked bar charts show the percentage distribution of overall image quality (**top**), luminal attenuation (**middle**), and diagnostic confidence (**bottom**).

All protocols received favorable ratings, with a range of excellent to good scores in terms of image quality. In this regard, no statistically significant differences were observed among the subgroups (median value 4 [interquartile range 4–4]; *p* ≥ 0.40, each). In contrast, luminal attenuation of ULC 55 keV (3 [2], [3]) was rated inferior to the other protocols, including ULC 45 keV (ULC 45 keV: 4 [4]; *p* < 0.010; SC: 5 [5]; *p* < 0.010; LC: 4 [4], [5]; *p* < 0.010). The attenuation of ULC 45 keV was deemed inferior to SC (p < 0.010) while no significant difference was observed in comparison to LC (p = 0.250). Exemplary CTA images comparing the image quality of all subgroups are shown in Fig. 3. The diagnostic confidence associated with ULC 45 keV was deemed good (4 [4]), though it was found to be inferior to the ratings assigned to SC (5 [5]; *p* < 0.001) and LC (5 [4], [5]; *p* = 0.026), exhibiting excellent results. However, ULC 45 keV exhibited superior performance in comparison to ULC 55 keV (3 [3], [4]; *p* < 0.001), yielding only moderate confidence. The ratings are summarized in Table 4 and visualized in Fig. 4. The interrater reliability was good for all categories, with each Kendall’s W ≥ 0.51.

**Table 4:** Subjective image quality.

	SC	LC	ULC 55 keV	ULC 45 keV
Overall image quality	4 [4]^1^	4 [4]^1^	4 [4]^1^	4 [4]^1^
Luminal attenuation	5 [5]^3^, ^4^, ^5^	4 [4], [5]^2^, ^4^	3 [2], [3]^2^, ^3^, ^5^	4 [4]^2^, ^4^
Diagnostic confidence	5 [5]^4^, ^5^	5 [4], [5]^4^, ^5^	3 [3], [4]^2^, ^3^, ^5^	4 [4]^2^, ^3^, ^4^

Values are given as median and 25%/75% interquartile ratio.

SC, standard contrast agent protocol; LC, low contrast agent dose; ULC, ultra-low contrast agent dose.

1 No significant differences between the groups (*p≥*0.05)

2 Significantly different compared to SC (*p* < 0.05)

3 Significantly different compared to LC (*p* < 0.05)

4 Significantly different compared to ULC 55 keV (*p* < 0.05)

5 Significantly different compared to ULC 45 keV (*p* < 0.05)

## 4 Discussion

This study retrospectively evaluated an ultra-low contrast agent protocol for photon-counting detector CT angiography in comparison to established low and standard contrast agent dose protocols. The findings indicate that, despite the ultra-low contrast agent protocol reducing the necessary contrast agent by half or two-thirds, respectively, 45 keV virtual monoenergetic reconstructions provide adequate contrast and image quality for reliable diagnostic evaluation.

A further reduction in the contrast agent dose to below 14.0 g, as utilized for the LC protocol, necessitated numerous protocol adaptations in prior studies. The utilization of the previous contrast agent (Imeron® 350 mgI/ml) and flow rate (4.0 ml/s) in conjunction with the reduction of iodine dosage to 7.2 g (equivalent to 20.6 ml) would result in a bolus length of approximately 5 s. This would consequently pose a relevant risk of missing this brief bolus during image acquisition. In order to generate a safe buffer, it was necessary to reduce the concentration of the contrast agent from 350 mgI/ml to 150 mgI/ml. This adaptation resulted in a comparable contrast agent volume (40 ml vs. 48 ml), despite a decrease in the total dose of iodine administered. A secondary adjustment was implemented, entailing a reduction of the flow rate from 4.0 ml/s to 2.5 ml/s. This adjustment resulted in an extension of the bolus length of the ULC protocol from 12.0 s to 19.2 s, thereby enabling homogeneous contrast agent application.

The pertinent advantages of the photon-counting detector technology to this study are as follows: firstly, the technology possesses the capacity for intrinsic higher weighting of low-energy photons, resulting in increased iodine contrast; and secondly, the capability to perform spectral imaging without increasing the radiation dose [8]. The latter facilitates the generation of VMI, which further augments iodine attenuation without exposing patients to additional radiation. In the context of (very) low-dose iodine imaging, as implemented in this study, both of these advantages are of particular significance in ensuring the acquisition of sufficient diagnostic images.

The analysis of CTAs acquired with ULC protocol settings and reconstructed at a VMI level of 45 keV revealed an intraluminal attenuation of approximately 300 HU in most cases. These studies were deemed adequate for diagnostic assessment, aligning with prior research [14], [15]. VMI reconstructions with a marginally elevated keV level of 55 keV resulted in a relevant proportion of examinations exhibiting an intraluminal attenuation of below 200 HU (11 out of 32), suggesting compromised diagnostic quality. Based on these results, we recommend a VMI level of 45 keV for implementing the ULC protocol, due to the improved objective and subjective image quality. Due to the increasing risk of non-diagnostic examinations, it is not recommended to further reduce the contrast agent dosage. This assertion is reinforced by three examinations with a luminal contrast of < 200 HU, which are rendered non-diagnostic in the presented ULC sample due to periprocedural errors. These included the incorrect placement of the trigger level (n = 2) and mixing effects in the periphery of a large aortic aneurysm (n = 1). The luminal contrast could not be increased beyond 200 HU and therefore diagnostic quality could not be attained, even in 45 keV VMI reconstructions.

The applicability of the ULC protocol is influenced by two major factors: large aortic aneurysms and cardiac comorbidities, particularly reduced cardiac output. The short bolus lengths of the ULC and LC protocols carry the risk of insufficient contrast due to delayed ejection of the contrast medium or dilution within an aneurysm. This results in a reduction of intraluminal contrast, particularly within the distal aorta and the pelvic arteries. Consequently, the ULC protocol has limited suitability for the initial diagnosis of an unknown pathology or for preoperative imaging, even though only one of 31 examinations was inadequate. In our opinion, the investigated ULC protocol is suitable for follow-up examinations to protect the renal function of patients with pre-existing renal diseases [16] due to comorbidities such as metabolic syndrome, diabetes mellitus, and medial sclerosis [3], [17].

In the context of contrast-agent induced nephropathy, studies have demonstrated that contrast agents with a lower osmolality and subsequent low viscosity exhibit a positive effect on renal clearance, hence protecting kidney function [18], [19]. The extant data in the literature indicates that contrast agents iso-osmolar to blood are the most optimal option [20], [21], [22]. Imeron® 150 mgI/ml, as utilized in the ULC protocol investigated in this study (osmolality of 301 ± 14 mosmol/kg at 37°C), is similar to the blood osmolality (275–295 mosmol/l) and possesses approximately half the osmolality of Imeron® 350 mgI/ml (618 ± 29 mosmol/kg) [23]. This characteristic renders it highly suitable for the intended purpose of protecting kidney function especially in patients with preexisting kidney disease or an increased risk for kidney injury. Concurrently, the decline in iodine concentration results in a viscosity reduction from 7.5 ± 0.6 mPa·s to 1.4 + /- 0.1 mPa·s, representing a decrease of approximately one-fifth. The enhanced renal clearance that ensues from this circumstance also serves to mitigate the risk of nephrotoxicity [24], [25].

In addition to its benefits for individual patients, a reduction in the amount of contrast agent has also been demonstrated to have an ecological impact. Iomeprol, the active ingredient in Imeron®, is excreted by the kidneys with minimal metabolic alteration. inconsequently, the presence of Iomeprol in wastewater and its potential accumulation in the environment due to its high molecular stability have given rise to concerns about possible negative biological effects [26].

Some limitations of this study must be acknowledged. Firstly, this was a retrospective, single-center investigation, was conducted with the only commercially available PCD-CT scanner at the time of research with its inherent limitations. Secondly, the study group was of a relatively small size. Thirdly, patients with ULC were continuously included in the study. Given that no obese patients were examined during the study period, obese patients were also excluded from the comparison groups, thereby introducing a certain selection bias with respect to body weight. Further studies with obese patients are necessary to evaluate the extent to which the results of the present study can be generalized. Fourthly, only contrast agents from a single vendor were applied in the respective scan protocols. However, transferability of results presented in this study to other contrast agents is assumed. Fifthly, we retrospectively reviewed protocols that were used in routine clinical practice. As a result, we did not have access to raw data for analysis, but only to the reconstructions typically used at our institute. Consequently, we were only able to analyze images using a soft reconstruction kernel (Bv36). The analysis of a solitary, pliant kernel represents a discernible constraint in the present study; nevertheless, it is intended to mirror clinical practice with the utmost precision. Studies have shown that sharper reconstruction kernels are associated with increased vascular sharpness, at the expense of increased image noise [27]. Future studies should examine whether these findings are applicable to protocols using ultra-low contrast agent doses. Finally, the 45 keV VMI were solely applied for the ULC protocol to offset the concomitant increase in image noise and the already sufficient intraluminal attenuation of the LC and SC protocols using 55 keV reconstructions.

## 5 Conclusion

Despite inferior contrast, aortic PCD-CTA with ultra-low contrast agent dosage at 45 keV still allows for diagnostic image quality. The proposed scan protocol may hold potential for kidney protection in vulnerable patient groups.

## Ethical Approval

The study was prepared in accordance with the Helsinki Declaration and was approved by the Institutional Review Board of the University of Würzburg (approval number 2025–125-dvhd).

## Author contributions

JLH and PG designed the study. JLH analyzed all data and prepared the manuscript. PG and JPG supervised the study. KK, HH, PP and VH performed observer analysis. KK, HH, PP, RSD and VH supported preparation of the manuscript and figures. JLH and PG performed statistical analysis. PG, JPG and VH revised the manuscript. TAB, HH, VH and JPG contributed to preparation of the manuscript and provided quality control. All authors read and approved the final manuscript.

## Informed Consent

The need for written informed consent was waived by the Institutional Review Board.

## Funding

Open Access funding was enabled and organized by Projekt DEAL and supported by the Open Access Publication Fund of the 10.13039/501100008769University of Würzburg. PG (Z−02CSP/18), PP (Z−2/CSP−33) and KK were financially supported by the Interdisciplinary Center of Clinical Research Wurzburg. The institution received financial support by Siemens Healthineers and the German Research Foundation.

## CRediT authorship contribution statement

**Kristina Krompaß:** Methodology, Investigation. **Jan-Lucca Hennes:** Methodology, Investigation, Formal analysis, Data curation. **Thorsten Alexander Bley:** Supervision, Funding acquisition. **Viktor Hartung:** Writing – review & editing, Validation, Supervision. **Philipp Gruschwitz:** Writing – review & editing, Project administration, Methodology, Funding acquisition, Conceptualization. **Pauline Pannenbecker:** Writing – review & editing, Methodology. **Henner Huflage:** Writing – review & editing, Validation. **Jan-Peter Grunz:** Writing – review & editing, Supervision, Methodology. **Ramin Saam Dazeh:** Writing – review & editing, Investigation.

## Declaration of Competing Interest

PG, PP and KK were funded by the Interdisciplinary Center of Clinical Research Wurzburg. JPG and TAB serve as research consultants for Siemens Healthineers. The Department of Diagnostic and Interventional Radiology receives research funding by Siemens Healthineers. The authors of this manuscript declare no further relationships with any companies, whose products or services may be related to the subject matter of the article.

## Data Availability

The datasets generated and/or analyzed during the current study are not publicly available but are available from the corresponding author on reasonable request.
