# Supplementary material for: Ultra-low contrast agent dosage in photon-counting CT angiography of the thoracoabdominal aorta
Source: Eur J Radiol Open. 2026 Jun 22;17:100777. doi: 10.1016/j.ejro.2026.100777 (PMC13316275; doi:10.1016/j.ejro.2026.100777)
Supplement: Supplementary file 1 — Supplementary material [file mmc1.docx]

**Supplementary Figure 1:**

**Exemplary ROI placement for measurements of intraluminal attenuation and in muscle tissue as well as standard deviation of attenuation in fatty tissue.**


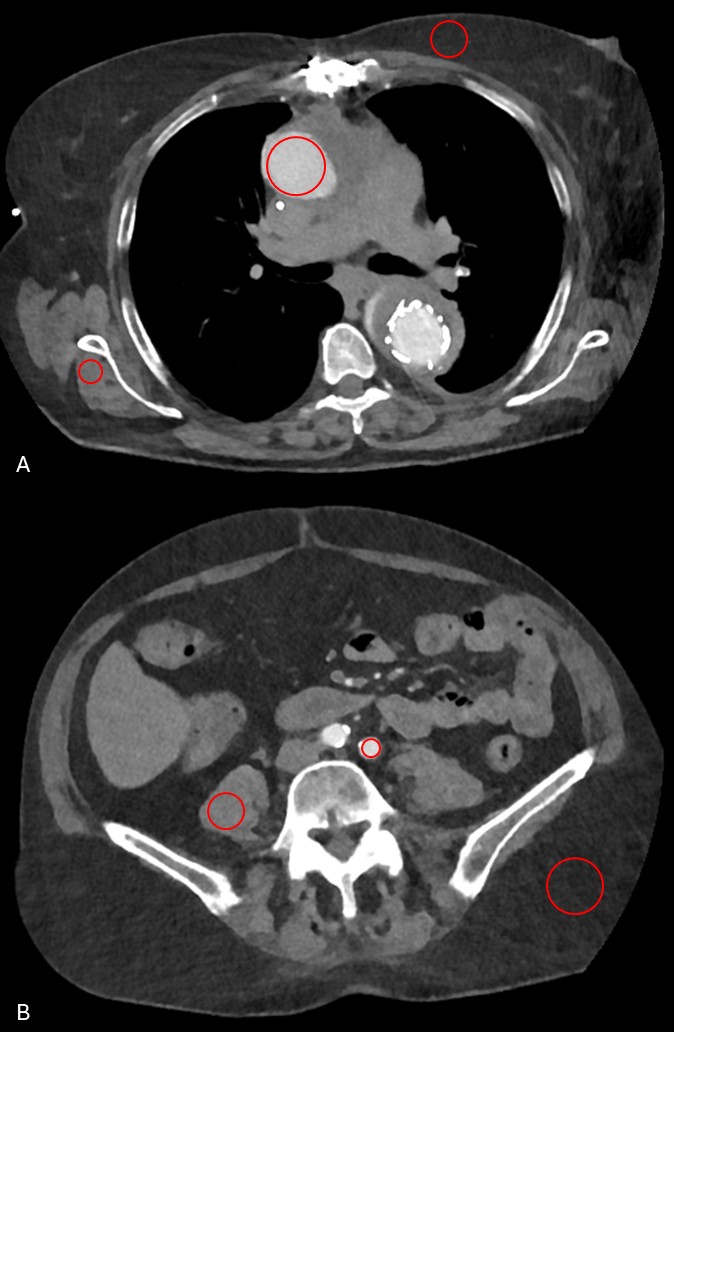


Sample images from a CT angiography performed on a photon-counting detector CT using a 45 keV ultra-low contrast agent dose protocol. Image A shows a sample measurement in the ascending aorta at the level of the pulmonary trunk, with measurements of attenuation in muscle tissue (the infraspinatus muscle) and the standard deviation of attenuation in prepectoral adipose tissue. Image B shows an example measurement in the left common iliac artery, with measurements of attenuation in the psoas muscle and the standard deviation of attenuation in gluteal adipose tissue.

ROI, region of interest.

**Supplementary Table 1:**

**Exact locations of ROI for measurements of intraluminal attenuation and in muscle tissue as well as standard deviation of attenuation in fatty tissue.**

| **Arterial lumen** | **Muscle** | **Standard deviation of fat** |
| --- | --- | --- |
| Ascending Aorta | Infraspinatus muscle | Pectoral |
| Aortic arch | Infraspinatus muscle | Axillary |
| Descending thoracic aorta | Erector spinae muscles | Ventrolateral |
| Descending abdominal aorta | Erector spinae muscles | Ventrolateral |
| Iliac commune artery | Psoas muscle | Gluteal |

ROI, region of interest.
